# Perceptions of pelvic floor dysfunction and rehabilitation care amongst women in southeast China after radical hysterectomy: a qualitative study

**DOI:** 10.1186/s12905-022-01687-0

**Published:** 2022-04-09

**Authors:** Yu-ting Lai, Ai-wu Lin, Zhi-hui Zheng, Ya-li Wang, Hong-hong Yu, Xin-yong Jiang, Li Ge

**Affiliations:** 1 School of Nursing, Fujian University of Traditional Chinese Medicine, No. 1 Qiu Yang Road, Shangjie, Minhou, Fuzhou, 350122 Fujian Province China; 2 The First Affiliated Hospital of Fujian Medical University, Fuzhou, 350005 China

**Keywords:** Pelvic floor dysfunction, Radical hysterectomy, Perceptions, Rehabilitation care, Qualitative study

## Abstract

**Objective:**

To investigate the perceptions of pelvic floor dysfunction (PFD) and rehabilitation care amongst women after radical hysterectomy and to explore ways to improve quality of care.

**Methods:**

Thirty-six women who underwent radical hysterectomy at a hospital in southeast China were enrolled via purposive sampling. Semi-structured in-depth interviews were conducted. The texts were analysed via qualitative content analysis.

**Results:**

Four themes were obtained: serious lack of knowledge, heavy psychological burden, different coping strategies and great eagerness to receive multiparty support on PFD rehabilitation care.

**Conclusion:**

The society and professional staff should strengthen health education on PFD. Professionals should offer education before and after surgery and actively provide rehabilitation consultation to promote the availability of rehabilitation to support women with PFD rehabilitation care. In addition, family-centred care is an important way to support women to return to normal life, and women's need for family support should be more actively expressed. Moreover, knowledge of medical insurance should be popularised, especially in rural areas in China.

## Introduction

Radical hysterectomy is one of the most recommended surgical procedures for gynaecological malignancies owing to its high treatment rate [1]. However, it substantially changes the overall structure of the pelvis, thereby unavoidably damaging pelvic floor tissues. The disruption of local nerve supplies and distortion of anatomical relationships in the pelvic floor after this operation may lead to the development of diseases related to pelvic floor dysfunction (PFD) [2, 3].

PFD is defined as a broad set of pelvic floor conditions such as urinary incontinence, constipation, pelvic organ prolapse, sexual dysfunction and chronic pelvic pain syndrome [4]. These conditions impose heavy physical and mental burden to women and greatly influence their daily lives [5] and psychological health [6] by negatively impacting their self-care activity and family and work relationships. A study reported that the prevalence of PFD in low- and middle-income countries is 25% [7]. A recent meta-analysis of adult Chinese women noted that the prevalence of urinary incontinence is 31.1%, and it has remained at a high level since 2005 [8]. Pelvic organ prolapse also affects 9.67% of urban Chinese women [9]. A study in the USA showed that 66.8% of participants are not aware of pelvic floor rehabilitation to address PFD [5]. For women who develop PFD after undergoing radical hysterectomy, rehabilitation therapy and care are often the primary choice in postoperative rehabilitation; this treatment modality can remarkably improve PFD and prevent its development [10]. Ideally, rehabilitation care for women with this condition should be implemented in the early postoperative period to ensure recovery from this condition, which can effectively restore pelvic floor function and reduce the incidence of PFD [11].

However, women’s perceptions of PFD considerably influence their choice and adherence to treatment and care [12]. Perception is to use self-cognition to explain sensory signals across multiple spatial and temporal scales, thereby leading to action [13]. Therefore, the perceptions of PFD and rehabilitation care are the key to the recovery of pelvic floor functions of women after radical hysterectomy. To the best of our knowledge, the perceptions of PFD and rehabilitation care amongst Chinese women after radical hysterectomy have not yet been explored. Therefore, this study aimed to investigate the perceptions of PFD and rehabilitation care amongst women who underwent radical hysterectomy to explore ways to improve quality of care.

## Materials and methods

This study was conducted at a provincial hospital in southeastern China from March 2019 to July 2019. Women who underwent radical hysterectomy were recruited via purposive sampling according to their education levels, i.e., junior secondary education (9 years), senior secondary education (12–13 years) and higher education (15–19 years) [14]. Eligible women were approached and invited to participate in the study by a senior nurse working in the gynaecological oncology department of the provincial hospital. The inclusion criteria were as follows: women who underwent radical hysterectomy, including open radical hysterectomy and laparoscopic radical hysterectomy; those who had no serious complications; those without psychological and mental illness; those who were fluent in Mandarin; and those who signed informed consent. Participants were excluded if they were unable to complete the interview because of insufficient interview time or were emotionally unstable during the interview. Enrolments of participants were determined by the principle of data saturation. This study was approved by the Ethics Committee of the First Affiliated Hospital of Fujian Medical University (FAHFMU [2018] No. 073).

Data were collected via semistructured individual interviews. A self-development interview guide consisting of nine questions about the perceptions of PFD and rehabilitation care was used for data collection (Table 1). Following the interview guide, a researcher (Y. L.) interviewed each participant face-to-face for 30–45 min in a separate and quiet room at the hospital. The interviewer noted the nonverbal behaviour of the participants. The entire interview was recorded using a digital audio recorder. Then, the researcher (Y. L.) transcribed the participants’ responses verbatim into Chinese text within 24 h after each interview. The texts were translated into English by Y. L. (a master student of nursing with bilingual skills) and L. G. (a Ph.D. in nursing from Sweden with rich experience in qualitative research).

**Table 1:** Interview guide: questions about the perceptions of PFD and rehabilitation care amongst women after radical hysterectomy

What do you know about female pelvic floor function?
If a woman has PFD (such as urinary incontinence), what do you think is the cause?
How does radical hysterectomy affect you?
What is your biggest concern after radical hysterectomy?
How do you think radical hysterectomy will affect pelvic floor function?
What will you do to treat PFD?
What do you think about the rehabilitation care of pelvic floor function?
How do you think rehabilitation care of pelvic floor function will affect you?
What are the factors that affect your rehabilitation care of pelvic floor function?

Data were analysed using the qualitative content analysis described by Graneheim and Lundman [15]. The analysis included manifest and latent content analysis as follows: (1) the text was read several times to obtain a whole sense of the participants’ perception of PFD; (2) the text content was divided into meaning units according to the meaning of expression; (3) the meaning units were compressed and extracted into codes; (4) differences and similarities between codes were compared, and then the sorted codes with commonality were grouped into categories, i.e., manifest content analysis; and (5) the research team fully considered the social and cultural contexts of the interview content, discussed the formed codes and categories and linked the underlying meanings to form themes, i.e., latent content analysis.

## Results

Thirty-six women who met the inclusion criteria participated in this study. The mean age of participants was 49 years (range 42 to 58 years). Amongst the participants, 12 had a low educational level, 12 had a middle educational level, and 12 had a high educational level. The place of residence included rural area (n = 20) and urban area (n = 16). Seven participants had PFD symptoms before surgery. Half of the participants developed PFD symptoms after surgery. Twenty-eight participants were in the acute postoperative period within 3 months of surgery, whilst eight were in the postoperative follow-up period for over 3 months after surgery (Table 2). Four themes were formed from the interview texts: (1) serious lack of knowledge, (2) heavy psychological burden, (3) different coping strategies and (4) great eagerness to obtain mutiparty support on PFD rehabilitation care (Table 3).

**Table 2:** Participant characteristics (n = 36)^a^

Variable	Value
Age, years	49 (42–58)
Educational level^b^	
Junior secondary education (9 years)	12 (33.3)
Senior secondary education (12–13 years)	12 (33.3)
Higher education (15–19 years)	12 (33.3)
Place of residence	
Rural area	20 (55.6)
Urban area	16 (44.4)
Occupation	
Farmer	9 (25.0)
Chef	1 (2.8)
Staff	4 (11.1)
Teacher	5 (13.9)
Trader	8 (22.2)
Lawyer	2 (5.6)
Freelance	4 (11.1)
Retirement	3 (8.3)
Household income, yuan/month	
< 2,000	2 (5.6)
2,000–5,000	16 (44.4)
5,000–10,000	10 (27.8)
> 10,000	8 (22.2)
Did PFD occur before surgery?	
Yes	7 (19.4)
No	29 (80.6)
Did PFD occur after surgery?	
Yes	18 (50.0)
No	18 (50.0)
Disease type	
Uterine sarcoma	2 (5.6)
Ovarian cancer	11 (30.5)
Endometrial cancer	11 (30.5)
Cervical cancer	12 (33.4)
Surgical approach	
ORH	5 (13.9)
LRH	31 (86.1)
Postoperative time, month	
≤ 3 (acute postoperative period)	28 (77.8)
> 3 ( postoperative follow-up period)	8 (22.2)

*PFD* pelvic floor dysfunction; *ORH* open radical hysterectomy; *LRH* laparoscopic radical hysterectomy

^a^Values are given as mean (range) or number (percentage)

^b^Classified according to the education statistic data in 2018[14]

**Table 3:** Perceptions of PFD and rehabilitation care amongst women after radical hysterectomy

**Serious lack of knowledge**
Limited knowledge of PFD
Limited knowledge of pelvic floor rehabilitation care
**Heavy psychological burden**
Fear and worry
Shame and self-contempt
Guilt and self-blame
**Different coping strategies**
Positive coping strategies
Passive coping strategies
**Great eagerness to receive multiparty support on PFD rehabilitation care**
Hope for receiving support from family members
Hope for receiving support from professionals
Hope for receiving support from society

### Serious lack of knowledge

The first theme consisted of two categories: ‘limited knowledge of PFD’ and ‘limited knowledge of pelvic floor rehabilitation care’.

#### Limited knowledge of PFD

Limited knowledge of PFD was reflected amongst most participants, regardless of whether she was in the acute or follow-up postoperative period. Most participants said that they were unaware of PFD: *‘I don't know what this pelvic floor disorder means, I don't understand’ (P14, postoperative period within 3 months, lived with PFD before and after surgery).* However, after learning the symptoms of PFD, some of the participants suggested that their advanced age and childbirth might have caused the disease: *‘The symptoms were normal. You know…so big surgery… It (nocturia) is not a disease. It needs to be slowly recovered. Do not need to see doctor for it’ (P22, postoperative period over 3 months, lived with PFD after surgery).* However, they were unsure if PFD was related to their radical hysterectomy: *‘I think a good doctor and very accurate surgery will not cause problems in patient’s pelvic floor function at all. I don't know whether previous surgery will cause this problem to women. I have never seen it before’ (P6, postoperative period within 3 months, lived without PFD after surgery).*

#### Limited knowledge of pelvic floor rehabilitation care

With regard to dealing with PFD, the participants did not know how to perform rehabilitation care, although some women recognised the efficacy of this treatment: *‘When I stand up, I feel that something in my abdomen will fall out, which should be related to the operation. I think the pelvic floor rehabilitation care may improve the situation, but I have no idea what I can do for it’ (P23, postoperative period within 3 months, lived with PFD after surgery).* Some of them even questioned its efficacy: *‘After this operation, my pelvic floor function was affected. The most obvious symptom is constipation. …I think pelvic floor function rehabilitation seems to be a psychological comfort. No actual effect. This organ has already been cut off, so physiological change cannot be changed’ (P15, postoperative period over 3 months, lived with PFD after surgery).* Several women said that they would not seek pelvic floor function rehabilitation care if they did not have symptoms of PFD: ‘*Vaginal delivery may cause the symptoms of PFD, like sexual dysfunction and urinary incontinence. However, if I do not have symptoms of PFD, I will not consider pelvic floor rehabilitation care unless I have such symptoms’ (P9, postoperative period within 3 months, lived without PFD after surgery).* Therefore, the participants were unaware of the importance of early rehabilitation care after surgery.

### Heavy psychological burden

The second theme was further categorised into ‘fear and worry’, ‘shame and self-contempt’ and ‘guilt and self-blame’.

#### Fear and worry

For the participants in the acute postoperative period, they felt more fear and were afraid. Some of them felt fear after experiencing PFD symptoms and were worried that they will no longer have a normal life: ‘*I fear, I fear that the urine cannot be closed…After I walk a little bit, urine can go itself. The smell…is bad (silence). My life is broken’ (P21, postoperative period within 3 months, lived with PFD after surgery).* Under the threat of malignant tumours, some women were afraid to obtain unfavourable information regarding their disease: *‘I do not want to ask anything (doctors)…Knowing too much will hurt me even more’ (P10, postoperative period within 3 months, lived without PFD after surgery).* Moreover, they were worried about their PFD symptoms worsening: *‘Of course, I am anxious. I am worried day and night that something will fall out of my body. And I always feel empty in my lower abdominal. This feeling kept me cannot fall sleep all night.’ (P7, postoperative period within 3 months, lived with PFD after surgery).*

#### Shame and self-contempt

As the postoperative time increased, the participants gradually paid attention to the distress caused by PFD. Stigma surrounding the problem of PFD contributed to participants feeling shame: *‘It (PFD) is a terrible thing. I will feel ashamed if I say it (silence). People will say something bad if they know it…’ (P31, postoperative period over 3 months, lived with PFD after surgery).* Moreover, they felt self-contempt because they lost their uterus, which meant that they lost their femininity, and they considered that they were no longer a full female: *‘The psychological burden is very heavy, and I am self-contemptuous that I do not be like a whole woman after the operation (silent), just like what they say’ (P2, postoperative period over 3 months, lived without PFD after surgery)*.

#### Guilt and self-blame

Furthermore, a few participants narrated that their family had spent a lot of money for their treatment. Despite the intervention, they did not fully recover and remained sick because PFD appeared after the operation. Hence, they felt guilty and blamed themselves: *‘After cost so much money on the disease, I would feel guilty if I am always a sick person after I go back home (silent). … I don't want my family to be worried about me too much’ (P13, postoperative period within 3 months, lived without PFD after surgery).* Several women apologised to their husbands for the shattered sex life, which limited their ability to fulfil marital roles: *‘Tell you the truth… I did the operation and now I have the disease (PFD) again…At this point, I'm sorry for him (husband), I can only let him do whatever he wants’ (P4, postoperative period over 3 months, lived with PFD after surgery).*

### Different coping strategies

The third theme presented two completely different coping strategies from participants to deal with PFD: one was ‘positive coping strategy’; the other was ‘passive coping strategy’.

#### Positive coping strategy

Most women with PFD were being positive. They described that they adjusted their mentality: *‘At the same time of treatment, I also adjust my mentality, should not be pessimistic. Even when facing a very serious matter, we must be optimistic and accept it’ (P12, postoperative period within 3 months, lived with PFD after surgery).* Moreover, they actively sought information regarding this condition, actively treated the disease and focused on their recovery. As one woman said, *‘First found it out from internet how to deal with it. Then, I went to the hospital for help…’ (P11, postoperative period within 3 months, lived with PFD after surgery).* Another woman pointed out, *‘I am doing whatever the doctor says. I rest more, exercise properly, and eat more for nutrition’ (P23, postoperative period within 3 months, lived with PFD after surgery).*

#### Passive coping strategy

However, one woman did not seek treatment and merely accepted her situation as ‘destiny’: *‘I think… Everyone has everyone's fate. It is destined, can't be changed… Look at me, my pelvis always feels a little pain and constipation occurs frequently, but I think it's nothing…It is better to let nature take its course instead of fighting against the fate… Accept the arrangements of fate’ (P25, postoperative period over 3 months, lived with PFD after surgery).* Some women only went to see a doctor when their symptoms started to affect their lives: *‘Well, if it doesn't affect my normal life and work, I usually don't go to the hospital, unless the symptoms of PFD are very serious’ (P29, postoperative period within 3 months, lived with PFD before surgery).* One woman completely stopped seeking help because of her advanced age: *‘My life is definitely getting worse because of the symptom (frequent urination) …I didn’t see doctor. Life is getting worse day by day if you are old. It doesn't matter’ (P1, postoperative period over 3 months, lived with PFD after surgery).*

### Great eagerness to receive multiparty support on PFD rehabilitation care

The fourth theme was great eagerness to obtain multiparty support for PFD rehabilitation care, which included ‘family support’, ‘professional support’ and ‘social support’.

#### Hope for receiving support from family members

All women hoped for understanding from their family members: *‘My family refused to pay the cost (silence)…They do not understand my suffering…’ (P18, postoperative period within 3 months, lived with PFD after surgery).* Although the participants gradually recovered with time, their energy was inevitably affected, and they hoped that family members could take some of their workload: *‘I have a shop…I still don’t have energy now… I have to work in my shop as soon as I am discharged from hospital. I need my family help me…’ (P13, postoperative period within 3 months, lived without PFD after surgery).* They also hoped to obtain decision support from their husbands in seeking rehabilitation and treatment: *‘I do not know whether my husband permit me see doctor or not. I will come if my husband let me come’ (P30, postoperative period within 3 months, lived without PFD after surgery).*

#### Hope for receiving support from professionals

Most of the participants indicated that professional support was necessary. They hoped that health-care staff could provide professional knowledge, skill support and care for them: *‘My friends and I never heard about it (PFD)… Doctor and nurse teach me how to exercise. I want to know more from them. I do want that they can teach me more and guide me more’ (P6, postoperative period within 3 months, lived without PFD after surgery).* Moreover, they stated that they would adhere to the health-care staff’s suggestions: *‘Leaking urine makes me feel embarrassed. I don’t dare to talk about it to others…Doctors and nurses are professionals. I especially hope that they can understand me and care me. I will at least relieve some of my anxiety’ (P16, postoperative period within 3 months, lived with PFD after surgery).*

#### Hope for receiving support from society

The participants also hoped to receive social support. One woman said that lack of money was a reason for her delay in seeking treatment, so she wanted to receive financial support from the government: *‘The cost of rehabilitation is too much for me… How can I still have money to go to the hospital for it, unless government gives me financial support’ (P4, postoperative period over 3 months, lived with PFD after surgery).* They believed that poor awareness of PFD in society played an important role when they sought support from society and even from family members: *‘Although I often read popular medical magazines, I was blank in this field before the doctor told me about the knowledge of pelvic floor dysfunction. I do think it is not known well by people in society. How can we get understanding and support from people around and society?’ (P26, postoperative period within 3 months, lived without PFD after surgery).*

## Discussion

This study presented that serious lack of knowledge played a major role that hindered early postoperative PFD rehabilitation care. Most participants stated inadequate knowledge about the meaning, cause of symptoms, effects and treatment of PFD, regardless of the postoperative period or whether or not they lived with PFD before and after the operation. On the one hand, it was may be because that all participants suffered from malignant tumour. They focused on treatment and surgical removal of the tumour whilst ignoring the impact of surgery on pelvic floor function. This observation was consistent with the rule of perception, i.e., humans focus on more important tasks in a complex environment [16]. In some participants’ view, malignant tumours also increased their fear of disclosing their condition and affected their initiative to obtain relevant knowledge about PFD and rehabilitation care. On the other hand, there is a huge knowledge gap of PFD amongst the public [17], which seriously influences the attitudes and behaviours of women and other people in their social circle toward rehabilitation care of PFD [18]. Most participants did not know that radical hysterectomy would result in PFD and described PFD as a ‘terminal illness’. The positive perceptions about pelvic floor rehabilitation could be not supported by a low level of public awareness about PFD prevalence and treatability. Therefore, health education about PFD and rehabilitation care is urgently needed. Participants pointed out that they hope to obtain relevant health information and rehabilitation guidance through professional channels. Therefore, knowledge of PFD and rehabilitation care should be popularised and strengthened in hospitals and society via multiple channels, such as brochures, posters, Internet, television and radio. Meanwhile, early postoperative rehabilitation can effectively restore pelvic floor functions and reduce the incidence of PFD [19]. Professional staff should grasp the opportunity of health education on PFD rehabilitation care in the hospital.

The present study indicated that amongst participants, losing their uterus meant losing their femininity, which made them reassess their female value and shame on the loss. They had to perceive themselves as a different person but not a normal woman [20]. The participants also feared the progression of their diseases and postoperative changes in their body. Postoperative PFD-related symptoms made them deny themselves and develop negative emotions, such as guilt and low self-esteem, which seriously affected their quality of life. This is similar to women who experienced pelvic floor trauma after vaginal birth [21]. A study showed that feelings of shame about the disease is the main factor why women do not see a doctor [22]. However, the women in the present study affirmed that they were eager to receive professional help. Therefore, health-care professionals should focus on the psychological and mental health of affected women, strengthen their confidence that they would recover and enhance their sense of self-worth whilst conducting health education/pelvic floor function rehabilitation care. In addition, an excellent intervention might be to organise a group of women who suffer from PFD with the assistance of health-care professionals. Doing so might alleviate their suffering and allow them to receive understanding and support from each other.

The interviews revealed that most of the participants adopted positive coping strategies in the face of PFD after radical hysterectomy. However, some of them adopted negative coping strategies, such as seeing a doctor only when the symptoms had affected their lives; gave up seeking help; and accepted their condition as their fate. Several reasons may explain these appearances. Firstly, some of them believed that PFD is an inevitable suffering brought by aging, and disease management is unnecessary in their old age. This belief was similar to that described by Vethanayagam et al. [23]. Secondly, the public’s misconceptions about PFD stigmatised women with this disease because of poor social awareness. The stigma reduces the possibility of women disclosing their illness and seeking help [24]. Thirdly, owing to the overarching influence of traditional Chinese concepts, some women in rural areas have a low degree of self-identity and largely rely on their husbands when it comes to making decisions about major family affairs [25]. As wives and mothers, women in traditional Chinese culture assume the responsibility as caregivers in the family, prioritising family obligations over their own health and hiding their own needs to decrease the burden on the family [26]. These reasons not only highlighted the importance of popularising PFD knowledge in society but also emphasised the importance of improving women’s self-identity.

An effective support system for individuals can reduce psychological burden, improve the level of disease response and ultimately promote disease recovery [27]. Family members usually promote disease recovery with economic, physiological, psychosocial and decision support. The participants hope that their families can share some tasks such as housework so that they can focus on PFD rehabilitation. All of them hoped to receive understanding and support from their families, especially from their husbands. Given that PFD is related to privacy and sex, understanding and support from husbands are especially important. The closer the family relationship is, the stronger the couple’s coping ability will be and the better they can deal with the problems caused by the disease [28]. Therefore, family-centred care is an important way to support women with PFD. Health-care professionals should focus on husbands, help women receive understanding and support from family members and encourage family members to actively participate in women’s rehabilitation care.

Moreover, the participants hoped to receive social support. Social support can not only promote patients’ healthy behaviours but also promote their compliance with medical regimens [29, 30]. This study shows that lack of treatment costs can delay participants in seeking treatment for PFD. Thus, they hope that the government can provide financial support for PFD rehabilitation care, such as including PFD rehabilitation in the basic medical insurance reimbursement to reduce the financial burden on women and increase their willingness to seek medical help. In addition, the medical insurance system in China covers all Chinese citizens living in the country. Thus, people can obtain preferential medical treatment at a relatively low price [31]. However, some women from rural areas said that they did not have medical insurance, suggesting that the public, especially in rural areas, lacks knowledge not only of PFD but also of medical insurance.

This study has several limitations. Firstly, the data may not be sufficient. The results might have been affected by some nuances in the translation of the texts from Chinese to English. Nevertheless, a qualitative study through detailed interviews and observations can provide insights into individuals’ thoughts, behaviour and understanding in life [32]. This study offered evidence on the low perception of PFD and rehabilitation care after radical hysterectomy amongst women and society. This study explored the underlying reasons and provided specific ways to improve the quality of care for women with PFD.

In conclusion, this study found that women who underwent radical hysterectomy in southeast China seriously lacked knowledge about PFD and rehabilitation care. Our data showed that women after radical hysterectomy bore a burden of physical discomfort and stigma because of inadequate information on PFD and rehabilitation care. The low awareness of PFD in society was also an important reason that women did not obtain more support from society and family members. Therefore, it is necessary to popularize PFD health knowledge in order to raise public awareness of PFD. Moreover, knowledge about pelvic floor rehabilitation should be imparted to women before radical hysterectomy. Professional staff should more actively inform patients about the symptoms of pelvic floor function damage that may be caused by radical hysterectomy, and actively provide patients with rehabilitation counselling before and after surgery. Professional staff should provide encouragement to patients to make them understand that they are there to provide help when symptoms of PFD worsen or persist. The knowledge of medical insurance should also be promoted in rural areas of southeast China. Family-centred care could be an important approach in the recovery of women with PFD. Women should be encouraged to express their needs for family support about postoperative recovery more broadly.

## Data Availability

The datasets used and/or analysed during the current study are available from the corresponding author on reasonable request.

## References

[1] Ebina Y, Mikami M, Nagase S, Tabata T, Kaneuchi M, Tashiro H (2018). Japan Society of Gynecologic Oncology guidelines 2017 for the treatment of uterine cervical cancer. Int J Clin Oncol.

[2] Laterza RM, Sievert KD, de Ridder D, Vierhout ME, Haab F, Cardozo L (2015). Bladder function after radical hysterectomy for cervical cancer. Neurourol Urodyn.

[3] Laterza RM, Salvatore S, Ghezzi F, Serati M, Umek W, Koelbl H (2015). Urinary and anal dysfunction after laparoscopic versus laparotomic radical hysterectomy. Eur J Obstet Gyn R B.

[4] Khatri G, de Leon AD, Lockhart ME (2017). MR imaging of the pelvic floor. Magn Reson Imaging Clin N Am.

[5] Burkhart R, Couchman K, Crowell K, Jeffries S, Monvillers S, Vilensky J (2020). Pelvic floor dysfunction after childbirth: occupational impact and awareness of available treatment. OTJR (Thorofare N J).

[6] Ghetti C, Skoczylas LC, Oliphant SS, Nikolajski C, Lowder JL (2015). The emotional burden of pelvic organ prolapse in women seeking treatment: a qualitative study. Female Pelvic Med Reconstr Surg.

[7] Islam RM, Oldroyd J, Rana J, Romero L, Karim MN (2019). Prevalence of symptomatic pelvic floor disorders in community-dwelling women in low and middle-income countries: a systematic review and meta-analysis. Int Urogynecol J.

[8] Kun Y, Zhenguo X, Zhihai Y, Sheng L, Meiping L (2019). Prevalence of urinary incontinence in Chinese adult women: a meta-analysis. Chin J Evid Based Med.

[9] Zhiyi L, Lan Z, Tao X, Qing L, Zhaoai L, Jian G (2019). An epidemiologic study of pelvic organ prolapse in urban Chinese women: a population⁃based sample in China. Natl Med J China.

[10] Arnouk A, De E, Rehfuss A, Cappadocia C, Dickson S, Lian F (2017). Physical, complementary, and alternative medicine in the treatment of pelvic floor disorders. Curr Urol Rep.

[11] Wenlin L (2017). Application of rehabilitation training in pelvic floor dysfunction after total hysterectomy. Mod Med Health Res Electron J.

[12] Tinetti A, Weir N, Tangyotkajohn U, Jacques A, Thompson J, Briffa K (2018). Help-seeking behaviour for pelvic floor dysfunction in women over 55: drivers and barriers. Int Urogynecol J.

[13] Jensen A, Merz S, Spence C, Frings C (2020). Perception it is: processing level in multisensory selection. Atten Percept Psychophys.

[14] Ministry of Education of the People ‘s Republic of China. Education Statistic Data in 2018.http://www.moe.gov.cn/s78/A03/moe_560/jytjsj_2018/qg/201908/t20190812_394239.html. Published 2019. Accessed August 8, 2019.

[15] Graneheim UH, Lundman B (2004). Qualitative content analysis in nursing research: concepts, procedures and measures to achieve trustworthiness. Nurs Educ Today.

[16] Boag RJ, Strickland L, Loft S, Heathcote A (2019). Strategic attention and decision control support prospective memory in a complex dual-task environment. Perceptions.

[17] Fante JF, Silva TD, Mateus-Vasconcelos E, Ferreira C, Brito L (2019). Do women have adequate knowledge about pelvic floor dysfunctions? A systematic review. Rev Bras Ginecol Obstet.

[18] Cox JH, Nahar A, Termine C, Agosti M, Balottin U, Seri S (2019). Social stigma and self-perception in adolescents with tourette syndrome. Adolesc Health Med Ther.

[19] Wenjun L, Limin F (2012). Study on correlation of time and pelvic floor dysfunction after hysterectomy. Natl Med Front China.

[20] Silva Cde M, Vargens OM (2016). Woman experiencing gynecologic surgery: coping with the changes imposed by surgery. Rev Lat Am Enfermagem.

[21] Skinner EM, Barnett B, Dietz HP (2018). Psychological consequences of pelvic floor trauma following vaginal birth: a qualitative study from two Australian tertiary maternity units. Arch Womens Ment Health.

[22] Nguyen A, Cheung A, Berg W, Lee W, Weissbart S, Kim J (2020). What's in a name? Choosing a title for a female pelvic medicine and reconstructive surgery clinic: a prospective questionnaire. Neurourol Urodyn.

[23] Vethanayagam N, Orrell A, Dahlberg L, McKee KJ, Orme S, Parker SG (2017). Understanding help-seeking in older people with urinary incontinence: an interview study. Health Soc Care Comm.

[24] Alshammari S, Alyahya MA, Allhidan RS, Assiry GA, AlMuzini HR, AlSalman MA (2020). Effect of urinary incontinence on the quality of life of older adults in riyadh: medical and sociocultural perspectives. Cureus.

[25] Zhongwu L, Qunyong W (2020). Psychological channel to improve women’s family status: a case of self-esteem. Stat Res.

[26] Chang L, Basnyat I (2017). Exploring family support for older Chinese Singaporean women in a confucian society. Health Commun.

[27] Jeong A, An JY (2017). The moderating role of social support on depression and anxiety for gastric cancer patients and their family caregivers. PLoS ONE.

[28] Checton MG, Magsamen-Conrad K, Venetis MK, Greene K (2015). A dyadic approach: applying a developmental-conceptual model to couples coping with chronic illness. Health Educ Behav.

[29] Barton C, Effing TW, Cafarella P (2015). Social support and social networks in COPD: a scoping review. COPD.

[30] Usta YY (2012). Importance of social support in cancer patients. Asian Pac J Cancer Prev.

[31] Yingfeng F, Chenyu Z (2020). The long-term effects of health on poverty reduction—the evaluation based on the new cooperative medical scheme. Mod Econ Sci.

[32] Denny E, Weckesser A (2019). Qualitative research: what it is and what it is not: Study design: qualitative research. BJOG.

